# Immunological Evasion in Glioblastoma

**DOI:** 10.1155/2016/7487313

**Published:** 2016-05-15

**Authors:** Roxana Magaña-Maldonado, Elda Georgina Chávez-Cortez, Nora Karen Olascoaga-Arellano, Mariana López-Mejía, Fernando Manuel Maldonado-Leal, Julio Sotelo, Benjamín Pineda

**Affiliations:** Neuroimmunology and Neurooncology Unit, The National Institute of Neurology and Neurosurgery (NINN), Insurgentes Sur 3877, 14269 Mexico City, DF, Mexico

## Abstract

Glioblastoma is the most aggressive tumor in Central Nervous System in adults. Among its features, modulation of immune system stands out. Although immune system is capable of detecting and eliminating tumor cells mainly by cytotoxic T and NK cells, tumor microenvironment suppresses an effective response through recruitment of modulator cells such as regulatory T cells, monocyte-derived suppressor cells, M2 macrophages, and microglia as well as secretion of immunomodulators including IL-6, IL-10, CSF-1, TGF-*β*, and CCL2. Other mechanisms that induce immunosuppression include enzymes as indolamine 2,3-dioxygenase. For this reason it is important to develop new therapies that avoid this immune evasion to promote an effective response against glioblastoma.

## 1. Current Status of GBM

Gliomas are the most frequent primary brain tumors in the Central Nervous System (CNS), with glioblastoma (GBM) being the most malignant tumor [1]. This tumor is characterized by great cellular heterogeneity, high invasiveness because of a large network of blood vessels, and ability to infiltrate healthy tissues. The National Institute of Neurology and Neurosurgery in Mexico reports that GBM represents 9% of all brain tumors and about 45.7% of gliomas [2, 3]. Current therapy may combine several and different approaches as surgery, radiotherapy, and chemotherapy, with alkylant agent temozolomide (TMZ) being used as standard treatment for GBM [4]. Despite this, the prognosis is still unfavorable with a median survival about 14.6 months [5, 6]. Diverse disciplines are developing strategies to improve current treatments; one of them involves immunological approach. This discipline represents an attractive alternative of therapeutic due to its less adverse effects, high selectivity, and ability to induce an effective immune response against the tumor.

## 2. Immune Response against GBM

Tumoral cells could be eliminated by the immune system in a process called immunological surveillance [7]. At the beginning, the thought was that brain tumors were separated from immunosurveillance, because they reside in an anatomical compartment lacking a normal lymphatic drainage system, so the CNS had been considered an immunological privileged organ with a very low level of T lymphocytes infiltration; however in pathological states, the lymphocyte trafficking increases because of the high permeability of a disrupted blood-brain barrier (BBB) [8–10]. During an infection, it was thought that adaptive immune response starts in the periphery stimulating T cells which are able to recognize any antigen, and, then, they migrated into CNS through cerebrospinal fluid [11]. Nowadays, a long-term resident population of CD8 T cells persisting in the brain though infection is over has been described. These cells remain in the tissue supporting themselves; this means that they do not require an antigen to avoid apoptosis by T cell selection process. So they are similar to other resident memory T cells in other tissues and also they do not return to systemic circulation [11].

It was initially reported the presence of T lymphocytes (CD4^+^ and CD8^+^) in both rat brain tumors induced by N-methyl-N-nitrosourea and human gliomas [12–14]. Brain tumors are characterized by immune infiltrate of dendritic cells (DC), macrophages, microglia and natural killer cells (NK), besides T lymphocytes, which are associate with tumoral elimination [14–18].

The most effective immune response against tumoral cells, is the cytotoxic response, being the main component, T-lymphocytes (CD8^+^). These cells are also called cytotoxic T-lymphocytes (CTL), which have an important role inducing the lysis of cancer cells [19]. CTL are able to recognize antigenic peptides through their T-Cell Receptors (TCR), being the response amplified by interaction with other immune cells, such as antigen presenting cells (APCs). These APCs process peptides and tumor-associated proteins, which are presenting to T lymphocytes via Human Leukocyte Antigen (HLA) molecules class I and II; besides, cancer cells could present through HLA class 1 on their surface [20]. This interaction as well as the presence of co-stimulatory molecules such as B7-1/2 induce the release of perforin and granzyme proteins and other cytokines such as *γ* interferon (IFN-*γ*) and tumor necrosis factor *α*/*β* (TNF-*α*/*β*) by CTL. Likewise, CTL proliferation is induced by T lymphocytes (CD4^+^), secreting cytokines as IFN-*γ* and IL-2, enhancing its anti-tumoral effect [21].

Microglia is formed by resident immune cells in the CNS which respond to signals triggered by brain damage, inflammation and the presence of foreign pathogens [22, 23]. Furthermore, they participate in pathological conditions such as neurodegenerative diseases and brain tumors [24].

Microglia is part of microenvironment that promotes the development of GBM. Brandenburg et al. described the important role of resident microglia for angiogenesis and tumorigenesis in gliomas, so microglia is definitely involved in tumor growth. Moreover, this study showed that depletion of microglia/macrophages correlates with a decrease in cell proliferation and angiogenesis and therefore a reduction in tumor volume [25].

Other studies describe that microglia has immunosuppressive activity through release of particular cytokines such as TGF-*β* and IL-10, increase of FasL, and inhibition of T cells activation. Getting a strong immunosupression, microglia is able to reduce the expression of MHC-II and CD80 and secretion of TNF-*α* [26–29].

Natural killer T (NKT) cells are a subpopulation of T lymphocytes, which are considered tumor cell killers; they produce antitumor molecules, such as Fas ligand (FasL), IL-4, IFN-*γ*, IL-13, perforin, and granzyme, that also promote lysis of tumor cells [30, 31].

## 3. Mechanisms of Immunosuppression

Local and systemic immunosuppression caused by GBM have negative impact on the treatment. Tumor microenvironment is comprised of multiple cell types, including tumor-associated parenchymal cells (microglia, neural precursors cells, peripheral immune cells, and vascular cells), which interact between them and promote tumoral growth [32]. Macrophages phenotype M2, T regulatory lymphocytes (Tregs), and myeloid-derived suppressor cells (MDSC) participate in this microenvironment, which actively infiltrate GBM and suppress T cell function [17, 18, 33–35].

Some CD4^+^ T cells express *α* subunit interleukin-2 receptor (CD25^+^), formerly known as T regulatory cells (Tregs; CD4^+^FoxP3^+^CD25^+^) [36–38]. Current knowledge is that Tregs could infiltrate tumors acting as cellular immunosuppressors and at the same time contributing to pathogenesis and tumoral progression [39]. In tumoral microenvironment, Tregs play a direct or indirect downregulation induction on T lymphocytes (CD4^+^ and CD8^+^) through diverse mechanisms: they can interact directly with DC to induce an immunosuppressor phenotype, avoiding the T lymphocytes reaction (CD4^+^ CD8^+^); thus, they promote tumoral cells survival; moreover, Tregs produce IL-10 and TGF-*β*, which block directly the effector T lymphocytes response inducing anergy [35, 40, 41]. Also, it has been described that Tregs low population increases the survival rate to induced brain tumors in animal models [18, 42]. Therefore, it is necessary to eliminate this subpopulation to achieve an effective immune response [35].

Tumor-associated macrophages (TAMs) frequently acquire a M2 phenotype; in patients affected by brain tumors, the presence of these cells has been associated with high-grade tumors and low survival rate [43]. TAMs are frequently related to neoangiogenesis and negative outcomes since they release metalloproteases, such as membrane type 1-matrix metalloprotease (MT1-MMP); these enzymes break off intercellular binding and allow glioma cells to invade the brain parenchymal. Also, glioma cells release substance that stimulates their overexpression through TLRs signaling [44, 45].

Another immunosuppression mechanism is carried out by Myeloid-derived suppressor cells (MDSC). These cells were initially found in tumor-induced hosts, being T-cell blastogenesis suppressors [46]. MDSC are phenotypically double positive to granulocyte and monocyte markers His48^+^/CD11bc^+^ in rats or Gr1/CD11b in mice. Human MDSC have been described in other neoplasms such as melanoma and renal cell carcinoma [47–49]; besides there is an increase of this subpopulation in GBM [29, 50]. MDSC use multiple mechanisms to suppress T lymphocytes function, such as essential amino acids catabolism as arginine or tryptophan by arginase I or indolamine 2,3-dioxygenase (IDO), respectively; also, they produce reactive nitrogen compounds and immunosuppressive cytokines as TGF-*β* [51–53]. In rats immunized with glioma cells, an increase in tumor infiltrating MDSC was observed; these cells generate a decrement in T lymphocytes, through nitric oxide production, inducing apoptosis [54]. Recently an association between MDSC and CD4^+^ effector memory T cells has been described, through surface receptor programed-death-1 (PD-1). This receptor is expressed on the surface CD4^+^ effector memory T cells, while their respective ligand, PD-1L, is upregulated on tumor-derived MDSC that induce T cells suppression [55].

Diverse immunomodulators and immunosuppressive factors are secreted by glioma cells, for example, interleukin-6 (IL-6) and colony stimulating factor-1 (CSF-1), which play an important role in Th2 response; this enhances its activity resulting in a less effective response against tumors [56, 57]. Other factors, such as prostaglandins, interleukin-10 (IL-10), and cyclooxygenase-2 (COX-2) have been described as part of the immunosuppressive tumor microenvironment [58]. Glioma cells also segregate transforming growth factor-*β* (TGF-*β*) that stimulates epithelial mesenchymal transition (EMT), thus contributing to extravasation and migration. Moreover, they provide a favorable microenvironment for angiogenesis and immunoevasion and induce immunosuppression by increasing Tregs and inhibiting dendritic cells (DC), cytotoxic T lymphocytes (CTL), and NK cells [59]. CCL2 is a chemokine secreted by gliomas; its function consists in Treg recruitment and migration [60]. On the other hand, CXCL10 raises proinflammatory IFN-*γ* expression that triggers CD4^+^ T lymphocytes release and promote tumor rejection [61]. Indoleamine 2,3-dioxygenase (IDO) is a cytoplasmic enzyme that participates in tryptophan degradation. Its expression by antigen presenting cells in lymph nodes enables T cell tolerance, in part due to induction and recruitment of Tregs [62]. During neuroinflammation, IFN-*γ* upregulates IDO expression by glial cells [63].

Furthermore, hypoxic microenvironment is another important factor that contributes to immunosuppression; it promotes the expression of genes involved in angiogenesis and proliferation [64]. Hypoxia activates STAT3 pathway as well as proteins which constitutes this immunosuppressive pathway. An important proangiogenic factor is the vascular endothelial growth factor (VEGF) [65, 66], as well as hypoxia-inducible factor 1-*α* (HIF1-*α*), which increases Tregs subpopulation [67–71]. Besides, STAT3 participates as a mediator for recruitment of microglia with a subsequent enhanced immunosupressive response [72].

**Figure 1:**
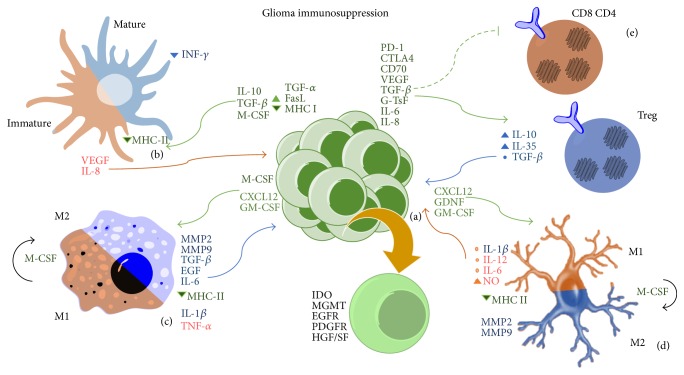
Diverse mechanisms used by glioma cells to generate immunosuppression. (a) Glioma cells secrete molecules that recruit regulatory T cells and inhibit cytotoxic T cells and Th1 lymphocytes proliferation. They promote the migration of MDSC and acquire an anti-inflammatory phenotype because of molecules like M-CSF. Glioma cells also increase receptors like EGFR and particular enzymes as IDO. (b) There is a predominance of immature DC and mature DC downregulate INF-*γ* expression. (c) The majority of macrophages population is represented by phenotype M2 which secretes MMP that remodel the extracellular matrix joined to other growth factors. (d) Phenotype M2 macrophages secrete MMP and different growth factors, supplying microglia infiltration. However, M1 profile does not have antitumor effect, because it generates cytokines such as IL-*β* inducing the expression of TGF-*β* by tumor cells. (e) Tregs downregulate other lymphocytes populations and are recruited by glioma.

Tumoral growth is associated with the release of microRNAs (miR). In glioma cells the expression of miR-92a generates tolerant natural killer T cells (NKT) and also promotes the expression of IL-10 and IL-6 in these cells, showing a reduction of perforin, Fas ligand, and interferon-*γ*. NKT cells can suppress cytotoxic CD8^+^ T lymphocytes [30]. Recently, we reported that specific changes on RB mutation and RAS overexpression in glioma cells confer properties to evade immune responses. These alterations enhance resistance to NK cell-mediated cytotoxicity [73]. Besides, NKT cells produce IL-13, which increase the expression of TGF-*β* through MDSC [74]. Some mechanisms are shown in Figure 1.

## 4. Therapeutic Strategies

Theoretically immune system would be able to generate tumoral eradication; however, this is limited due to multiple immunosuppressive mechanisms. Nowadays, there are diverse studies focused to eliminate this tumor-associated immunosuppression and promote an effective immune response against cancer [75, 76].

Several strategies are under investigation in order to reduce immunosuppression mediated by Treg cells [77]. Curtin et al. used PC61 and anti-CD25^+^ antibody at an orthotopic GBM murine model and they observed a depletion of Tregs cell population in different tissues such as tumor, lymph nodes, and spleen; besides a better long-term survival after systemic depletion of regulatory T cells was achieved. Remarkably, this improvement depends on tumor burden because no effect was seen trying to induce Tregs depletion 24 days after implantation, suggesting that it could be useful in minimal residual disease [18].

Recently, we reported the use of pertussis toxin (PTx) as adjuvant immunotherapy in a C6 glioma model, showing a decrease in tumoral size, selective cell death in Tregs, and less infiltration of tumoral macrophages [78]. In another study, we evaluated the cytotoxic effect of PTx in combination with temozolomide (TMZ) for glioma treatment, both* in vitro* and* in vivo* RG2 glioma model. We observed an induction of apoptosis in around 20% of RG2 cells, in both single treatments PTx and TMZ and their combination. Also, the treatment with PTx increases the formation of autophagy vesicles. Survival increased after individual treatments, and this effect was enhanced with the combination TMZ+PTx. Treatment with PTx reduced the number of Tregs in tumor. PTx could be an immunotherapeutic adjuvant in the integral therapy against GBM due to their multiple properties either directly in glioma cells or modulating immunological subpopulations. We demonstrated that its combination with TMZ could represent an advantage to improve the GBM treatment [79].

In a murine glioma model, TMZ treatment and vaccination with monoclonal antibody against IL2R*α* (CD25) showed a decrease of tumor growth as well as depletion of Treg cells without affecting the functions of effector T cell. They also demonstrated that administration of anti-CD25^+^ antibody in patients with glioblastoma reduced about 48% Treg population and raised an expansion of effector T cells induced by vaccination with DC directed to human cytomegalovirus antigen pp65 [4].

A recent study used both anti-CD25^+^ monoclonal antibody, daclizumab, and epidermal growth factor receptor variant III (EGFRvIII) vaccination in patients previously treated with temozolomide. Daclizumab reduced significantly the prevalence of circulating Tregs compared to control, without evidence of adverse effect in effector T cell response. Moreover, a greater EGFRvIII specific humoral response was observed when Tregs population was low, suggesting that this depletion may enhance vaccine-induced immunity [80].

PD-1 is located on the lymphocyte's membrane and is associated with immunosuppression in several tumors including GBM [81]. Anti-PD-1 immunotherapy was evaluated along with stereotactic radiosurgery in a mouse intracranial GBM model. Using the combinatorial therapy long-term survival as well as increased tumor infiltrating cytotoxic T cells and decreased regulatory T cells were seen [82].

Wainwright and colleagues researched the relevance of IDO expression by glioma cells, finding a better prognosis in patients with glioblastoma while IDO was downregulated. They also shown that mice with IDO-deficient brain tumor presented higher survival rate associated with a depletion of resident Tregs into the brain [83].

STAT3 inhibition offers a potential strategy to downstream immunosuppressive effects of tumor-associated microglia. Zhang et al. used a siRNA-based method to block STAT3 pathway in the GL261 model of murine glioma, resulting in a high activation of microglia/macrophage within tumor and improving clinical implications [84]. Inhibition of intratumoral STAT3 activity can also be achieved through delivery of miR-124 [85].

If microglia is activated and phagocytic activity is on top, a selective delivery of targeted agents could be developed. It has shown that one of the properties of carbon nanoparticles is increasing uptake of CpG oligonucleotides by murine macrophages/microglia. Both CpG oligonucleotides and CNP were injected intratumorally and resulted in improvement of survival period in the GL261 model [86].

## 5. Conclusion

It is clear that immunological therapies are important therapeutical alternatives in management of brain tumors, since an effective immune response could be able to eliminate neoplastic cells. Access of chemotherapy agents to glioblastoma is limited because of the existence of the blood-brain barrier; however, few immune system cells possess the ability to cross and infiltrate the tumor representing an advantage in comparison with antitumoral drugs. In glioblastoma there is no effective elimination of tumoral cells due immunomodulation that exerts these cells, creating a microenvironment predominantly immunosuppressor that allows tumoral proliferation. This review offers a general overview of some therapeutical strategies developed with the purpose of changing this immunosuppressor phenotype as well as avoiding the migration of immunosuppressor cells to tumor.
